# Exploratory studies to inform full-scale evaluations of complex public health interventions: the need for guidance

**DOI:** 10.1136/jech-2017-210414

**Published:** 2018-07-20

**Authors:** Laurence Moore, Britt Hallingberg, Daniel Wight, Ruth Turley, Jeremy Segrott, Peter Craig, Michael Robling, Simon Murphy, Sharon Anne Simpson, Graham Moore

**Affiliations:** 1 MRC/CSO Social and Public Health Services Unit, University of Glasgow, Glasgow, UK; 2 Centre for the Development and Evaluation of Complex Interventions for Public Health Improvement, School of Social Sciences, Cardiff University, Cardiff, UK; 3 Specialist Unit for Review Evidence, Cardiff University, Cardiff, UK; 4 Centre for Trials Research, Cardiff University, Cardiff, UK

**Keywords:** health behaviour, methodology, prevention, randomised trials, research methods

Addressing complex public health problems, such as smoking, obesity and mental health, requires complex, often multilevel, interventions. Given the costs associated with delivering such interventions and the possibility of unanticipated harm, they need to be evaluated using the most robust methods available. It is important, where possible, that public health interventions and their proposed evaluation designs are optimised prior to being subject to an expensive evaluation of their effectiveness, through rigorous assessment of their feasibility.^1^ Consequently, a growing number of exploratory studies (ie, studies intended to generate the evidence needed to decide whether and how to proceed with a full-scale evaluation, also (inconsistently) referred to as ‘pilot’ or ‘feasibility’ studies) are being conducted. These generally have one, or both, of the following objectives: to optimise or assess the (1) feasibility of the intervention or (2) design of the full-scale effectiveness evaluation. However, conflicting guidance exists regarding what exploratory studies should be called, what they should achieve, what they should entail, whether and how they should determine progression to future studies and how they should be reported.^2 3^ This presents a challenge for researchers in designing and conducting exploratory studies, and for peer reviewers and funders in judging the merits of research proposals and outputs. This paper briefly discusses these issues, before describing current work funded by the Medical Research Council (MRC)/National Institute of Health Research (NIHR) Methodology Research Programme to develop GUidance for Exploratory STudies of complex public health interventions (henceforth referred to as the GUEST study).

There is increasing recognition that pressure to identify effective interventions has led to premature commissioning of large-scale evaluation trials of poorly developed interventions, wasting finite resource.^1 4^ In the development of pharmaceuticals, over 80% fail to reach ‘Phase III’ effectiveness trials, even after considerable investment.^5^ Yet, with public health interventions there has been a tendency to rush to full evaluations, which commonly fail due to issues which could have been identified at a feasibility stage, such as the acceptability or feasibility of the intervention or difficulties recruiting or retaining participants. The importance of establishing the feasibility of the intervention and evaluation plans prior to embarking on an expensive, fully powered evaluation study was indicated in the MRC Guidance on the Development and Evaluation of Complex Interventions.^6 7^ In more recent years, major funders, such as the NIHR Public Health Research Funding Programme, have begun to fund a large number of pilot and feasibility studies, while the MRC has established the Public Health Intervention Development funding panel, increasing the volume of pre-evaluation research in public health. This has facilitated a shift towards an expectation that exploratory studies will address feasibility issues before funding a full-scale evaluation will be considered, including assessing the extent to which these issues are context dependent and the identification of an intervention’s mechanisms of change.^8 9^

Nevertheless, the extent to which substantial investment in exploratory studies has to date improved the effectiveness and cost-effectiveness of evidence production processes remains to be firmly established. Recently funded exploratory studies demonstrate that they can be costly (often between £300 and £500 k).^10^ If conducted poorly, this investment may lead to significant extra expenditure and several years’ delay in generating evidence, without necessarily increasing the likelihood that future evaluation will produce useful evidence. Examples are emerging of interventions which were assessed through a substantial feasibility and piloting phase, but still reported substantial problems with intervention implementation or study recruitment and retention at full evaluation stage. On the other hand, the imperative to test every potential uncertainty prior to full evaluation may have in some cases gone too far, with exploratory studies testing issues for which prior literature indicates minimal uncertainty. For example, the feasibility of randomisation in school-based interventions has been widely demonstrated. Nevertheless, many exploratory studies of school-based interventions continue to randomise, serving no purpose other than re-establishing (at great cost) that which we already know to be feasible. In such cases, studies could be more efficient through testing the intervention in all included schools to understand implementation in a wider range of contexts, or simply by not recruiting an unnecessary control group, reducing study cost. Finally, the suitability of exploratory studies to achieve certain aims may depend on their design. For example, use of exploratory studies to understand mechanisms may take the form of in-depth qualitative work or assessment of impacts on immediate proximal outcomes on the hypothesised causal pathway (provided there is power to do so).

The MRC guidance of 2000 used the term ‘exploratory trial’ for the work immediately prior to a ‘definitive trial’. In addition to evaluating key design components like recruitment and retention, it argued that an exploratory study should address issues concerning the optimisation, acceptability and delivery of the intervention. Not all research funding bodies, however, have as inclusive a concept of the potential goals of exploratory trials within their published definitions. For instance, the NIHR Evaluation Trials and Studies Coordinating Centre’s definitions of feasibility and pilot studies do not include any examination of intervention design, delivery or acceptability, nor do they suggest that modifications to the intervention prior to full scale evaluation will arise from these phases (http://www.netscc.ac.uk/glossary/). However, the NIHR portfolio of funded studies includes various terms such as ‘feasibility trial’, ‘pilot trial’ and ‘exploratory trial’ to describe funded studies with similar aims. In practice, such studies are rarely limited to exploration of methodological uncertainties, to the exclusion of uncertainties relating to intervention implementation.^11^

Guidance for exploratory studies has often been framed as relevant only where the main evaluation is to be a randomised trial.^7 12^ Previous MRC guidance in 2000 focused solely on randomised controlled trials, although updated MRC guidance in 2008 moved away from this, reflecting the recognition that for some public health interventions, randomised trials are not feasible or appropriate and rigorous non-randomised alternative designs, including natural experiment evaluations, are required. This highlights a possible gap in guidance for exploratory studies that might progress to non-randomised evaluation designs. In natural experimental evaluations, for example, the researcher will usually have no control over the intervention, while the time-sensitive nature of such work may sometimes force evaluators to go straight to evaluation without assessment of feasibility. However, where possible, exploratory studies in preparation for natural experimental evaluations are, nevertheless, important for addressing uncertainties about intervention components and their underlying logic, informing identification and selection of appropriate outcome measures and identifying implementation uncertainties to be addressed through process evaluation at the full evaluation stage. Addressing these gaps in the literature and providing effective guidance on exploratory studies will help ensure that the most promising interventions are evaluated in a timely fashion using the most feasible, rigorous designs, minimising expenditure of scarce resource on poor-quality evaluations of underdeveloped interventions.

## Overview of GUEST study

In developing new guidance, our study will examine current practice and expert consensus in relation to uncertainties that should be addressed prior to full evaluations of complex public health interventions, methodological considerations in addressing these uncertainties and decision-making regarding whether and how to progress to evaluation of effectiveness following the conduct of an exploratory study. The guidance is being developed through activities which include: (1) a systematic review of guidance on exploratory studies; (2) an audit of current practice; (3) a web-based DELPHI exercise to identify expert consensus on the purpose, design and conduct of exploratory studies involving consultation with stakeholders and (4) a horizon scan of novel approaches to intervention optimisation and exploratory study designs from other contexts within and outside health research. Guidance will not be limited to exploratory studies of a specific design and will assist researchers in public health to more efficiently and effectively develop and conduct exploratory studies, while providing peer reviewers and research funders with objective criteria against which to assess the quality of bids and publications. This will likely have substantial impacts on the health and well-being of the population and on the effectiveness and efficiency of preventive services and policies, by increasing rigorous and reliable evidence of what does and does not work.
